# From Mice to Men: How B Cell Immunology Helped the Understanding of Leukemia Development

**DOI:** 10.3389/fimmu.2018.02402

**Published:** 2018-10-24

**Authors:** Paolo Ghia

**Affiliations:** IRCCS Ospedale San Raffaele, Università Vita-Salute San Raffaele, Milan, Italy

**Keywords:** B cells, immunoglobulins, B cell development, B cell receptor (BCR), chronic lymphocytic leukemia

The work on normal B lymphocytes, immunoglobulins, and antigenic stimulation performed at the Basel institute in the early ‘90s, also by Anton G. Rolink, shaped and molded the way B cell lymphomas and in particular Chronic Lymphocytic Leukemia (CLL) are interpreted today.

Words like *Immunoglobulin rearrangements, somatic mutations*, and *B Cell Receptors*, which 25 years ago were of interest only to basic immunologists like Ton, became common and trivial (if ever possible) terms also in hematological and clinical gatherings. Nowadays immunologists are in fact regularly invited to such meetings as key-note speakers to explain the basic functionality of the immune system, which has direct implications on the daily clinical practice.

During that pioneering period we also learnt that animal models were not merely a surrogate tool for human organisms, necessary only for obvious reasons of simplicity and convenience, but on the contrary that they may provide precise insights that could be directly applied to the human system. This turned out to be true in the case of the maturation process of B lymphocyte progenitors and precursors in the bone marrow, as we demonstrated that the developmental ordering of the different stages of differentiation in human bone marrow were superimposable to those described by Ton years earlier in mice, with only few small but potentially interesting differences in phenotypic markers (1, 2). In light of such evidence, additional observations on B cell aging in mice and men have also been made, pointing to a progressive decrease of B cell production with age in both organisms (3) and to a restriction of the diversity of the repertoire, as underscored by the appearance of clonal B cell populations consisting of plasma cells or mature (mainly CD5^+^) B cells in the spleens of aged mice (4). Monoclonal Gammopathy of Undetermined Significance (MGUS) a premalignant phase for Multiple Myeloma (5) typical and common in the elderly, can be considered the human counterpart of murine monoclonal plasma cell expansions. At that time, no expansions of CD5^+^ mature B cells were known in aging individuals. In a subsequent publication (3), we initially suggested that CD5^+^ B cell clonal populations present in old mice might be considered analogous to precursors of Chronic Lymphocytic Leukemia (CLL), the most frequent B cell neoplasm in the elderly (6), which is characterized by clonal expansion of CD5^+^ B cells and for which no pre-leukemic phase was defined.

Few years later, such reasoning justified a large observational study involving hundreds of healthy elderly individuals in a quest for clonal expansions of mature B cells using high-sensitive flow cytometric techniques, not in spleens or lymphoid tissues as in mice but in the best surrogate available in humans i.e., the peripheral blood (7). This led to the discovery of either CD5^−^ or CD5^+^ monoclonal B lymphocytes in almost 10% of individuals above 40 years of age with an increasing frequency associated with age, peaking at an impressive 50% in healthy individuals aged more than 90 years (8). Similar observations became at the same time available from the UK (9), Spain (10), and the US (11, 12), suggesting the universality of the phenomenon that has been later defined by an international consortium as Monoclonal B cell Lymphocytosis (MBL) (13), potentially involving either CD5^−^ or CD5^+^ B lymphocytes. CD5^+^ MBL is the most frequent, accounting for two thirds of all MBL, and is characterized by the presence of monoclonal B cells with a phenotype identical to CLL (CD20^dim^, CD5^+^, IG^dim^) in the peripheral blood, at concentrations lower than those required for the diagnosis of CLL (5 × 10^9^/l) (13). Later on, we reported that MBL can be further distinguished into “low-count” MBL (LC-MBL) and “high-count” MBL (HC-MBL), based on the number of circulating CLL-like cells (more or less than 0.5 × 10^9^/l, respectively) (14). The former does not virtually progress into a clinically relevant disease and may be considered an interesting model to study B cell aging (15), while the latter may evolve into a clinically relevant CLL at a rate of 1% per year (9, 16) and may be considered a real pre-leukemic phase of CLL (12), with all the obvious clinical and pathogenetic implications. All these findings recently provided ground for the inclusion of MBL as a new disease entity within the classification of lymphoid neoplasms by the World Health Organization (WHO), similarly to MGUS (17). Nowadays, there are patients around the world diagnosed and followed-up because of the presence of MBL.

While MBL showed us, thanks to cross-fertilization between mouse and human immunology, how discoveries can sometimes be predictable, the progress of science remains unpredictable in other instances. This is best exemplified by Immunoglobulin genes, a unique exception to the gene-protein dogma in genetics because of the rearrangement process and the introduction of mutations, making them probably one of the most enlightening models of nature adaptation but also the least digestible concept to medical students and alike. The first study to show the developmental order of B progenitor and precursors of the B lymphocytes in the human bone marrow, performed at the Basel Institute under Ton's supervision, was entirely based on the description of the rearrangement status of the Immunoglobulin Heavy and Light chain gene loci, assessed at single cell level (2). In a pre-internet era, the gene sequences for all IG loci could only be collected one by one from printed text books and volumes and needed to be manually curated and aligned, a work that nowadays can be done online in a click. At that time, all that work appeared frustratingly far from the possibility of a concrete application in medicine. A few years later, that knowledge unexpectedly became crucial to better understand the pathogenesis of CLL, following the seminal observation that the presence of somatic mutations within the rearranged clonotypic IGHV genes associated with a better clinical outcome and prognosis in CLL patients (18, 19). This originated from the analysis of somatic hypermutation in neoplastic B cells to track back the cell of origin of mature and immature B cell neoplasms. Strangely enough, CLL, the easiest leukemia to diagnose with a typical and unique phenotype, was indeed a mixture of cases with and without somatic mutations within the IGHV genes, which puzzled all hematologists (and immunologists alike) (20). This observation fueled endless discussions and experiments aiming at clarifying the distinct origin of these 2 subsets of CLL while trying to explain the remarkable uniqueness of the surface phenotypic appearance (21). Only more recently several studies demonstrated that indeed the presence of somatic hypermutations was associated with deeper and more prolonged responses to immunochemotherapy, in particular to the combination of fludarabine, cyclophosphamide, and rituximab (FCR), the gold standard for the treatment of young, fit CLL patients. IG-mutated patients experience long term remissions reaching a plateau on the PFS curve, with no relapses beyond 10 years, an unprecedented finding in a disease that is still considered incurable (22–24). This transformed the IGHV gene mutational status from a prognostic marker in CLL into a predictive factor, allowing for a better upfront selection of the patients who are more likely to benefit from the use of immunochemotherapy. Testing for IGHV gene mutations is now recommended in the recent update of the guidelines by the International Workshop on CLL (iwCLL) (25), where it is stated that the determination of the somatic hypermutation status of the IGHV genes should always be performed before deciding the treatment of a patient with CLL, as it has relevant implications on the choice of the most effective treatment. This is now probably one of the most prominent examples of personalized medicine approaches that is envisioned in many types of cancer but that in CLL has become reality. A validated consensus methodology for reliable clinical-grade analysis of IGHV genes has been established by the European research Initiative on CLL (ERIC) and moved out of research laboratories into diagnostic facilities (26).

From a scientific point of view, IG gene analysis also became central in the study of CLL pathogenesis and culminated with the creation, as part of an international consortium (IgCLL, www.igcll.com), of a world-wide database collecting to date over 30.000 IG sequences from CLL patients. This remarkable collection helped identify and consolidate the unexpected finding that different unrelated CLL patients share very similar if not identical sequence motifs within the—variable heavy chain complementarity determining region 3 (VH CDR3), thus defining “stereotyped” B-cell receptor (BcR) (27). This made it possible to group at least 30% of CLL patients into subsets with similar BcR that, besides similar immunogenetic features, also share similar molecular and functional features and clinical prognosis beyond the simplistic dichotomy of mutational status (28). From an immunological point of view, the presence of stereotypy suggests the recognition of common structures, thus implying that CLL ontogeny is not stochastic but rather driven by interactions between the clonogenic cells and a restricted set of antigenic elements (27).

Thanks to all these molecular findings, it became evident that immunoglobulins and in particular the entire B cell receptor are central players in CLL pathogenesis, even overshadowing in some occasions the role of genetic aberrations. At variance with many other B cell neoplasias characterized by a single distinct genetic abnormality, a number of gross genomic abnormalities including deletion 11q, 13q, 17p, and trisomy 12, are found in different proportions of CLL cases (29). Moreover, high-throughput studies have revealed a further remarkable genetic heterogeneity, with certain genes (*NOTCH1, SF3B1, TP53*) mutated in only 10–15% of patients (30–32) and others mutated at even lower frequencies (e.g., *NFKBIE, RSP15, EGR2*) (33). No unifying genetic mechanisms or lesions have thus been identified insofar. On the contrary it has been demonstrated that the signaling pathway downstream of the BcR is active in all cases of CLL, regardless of the mutational status and immunogenetic features of the patient (34). This is particularly true in the leukemic cells obtained from lymph nodes, where CLL cells are believed to encounter the relevant antigenic elements in the context of the so-called *proliferation centers*, considered the reservoir of the disease: there, leukemic cells get to proliferate and expand also thanks to additional signals originating from T cells, stromal cells and monocytic-derived cells, or through other immune receptors such as the TLRs.

In a proportion of cases, a number of foreign or auto-antigens (35, 36) have been identified as able to interact and stimulate the leukemic BcR, in particular neoantigens, newly exposed in the context of apoptotic bodies produced during physiological cell turnover. However, the notion that classic auto-antigen recognition and binding is important for CLL ontogeny has been challenged by the recent demonstration of cell-autonomous signaling, a novel type of signal generation, occurring specifically in CLL amongst B cell lymphomas, through self recognition as a result of the interaction of the leukemic BcR IG with a conserved epitope of the same or adjacent BcR IGs (37). Autonomous signaling has first been demonstrated in B cell precursors where the pre-BCR has evolved to ensure self-recognition, allowing for positive selection at the pre-B cell stage (38). Interestingly, different portions of the Immunoglobulin are recognized by different stereotyped receptors, each with affinities that appear to associate with distinct clinical outcomes and/or biological responses (39). This heterogeneity resembles the long-known evidence that antigen-dependent stimulation in CLL may also be pleiotropic and may associate with clinical outcome, ranging from full activation and proliferation to anergy and survival (40). The former appears to be typical of more aggressive CLL, particularly with unmutated IGs, while the latter associates with more indolent cases, usually with mutated IGs, thus providing a functional basis for the clinical heterogeneity of the disease. It still remains to be elucidated how antigen-dependent and autonomous signaling cooperate in the onset and maintenance of CLL.

Despite the molecular evidence of the centrality of the BcR in CLL, the final proof that the BCR stimulation is crucial in all cases of CLL, regardless of the differences in mutational status, antigenic affinities or strength of autonomous signaling, has come from the impressive and virtually universal efficacy of the therapeutic inhibition of the BcR signaling pathway in CLL patients.

The accumulating knowledge of the biology and immunology behind CLL has been applied to the research for innovative therapies, leading to the design and approval of novel mechanism-based drugs such as ibrutinib (41) and Idelalisib (in association with the anti-CD20 antibody Rituximab) (42), targeting molecules downstream the BcR, namely BTK and PI3Kδ. Both drugs have shown greater efficacy and better tolerability than chemoimmunotherapy and have even dramatically changed the prognosis of high-risk CLL patients, including those with *TP53* aberrations.

Thanks to the immunological knowledge (and human wisdom) that has developed at the Basel Institute also thanks to Ton Rolink, all physicians may find themselves less at loss in a changing world of hematology where immunology has become central for understanding neoplastic diseases to the benefit of the patients. As unexpected as this was, it has become reality and it should encourage us all to be in part immunologists if we want to better understand the pathogenesis of leukemias and lymphomas. And we should all teach future generations to do so with fun every day!

## Author contributions

The author confirms being the sole contributor of this work and has approved it for publication.

### Conflict of interest statement

The author declares that the research was conducted in the absence of any commercial or financial relationships that could be construed as a potential conflict of interest.

## References

[1] ten Boekel E Melchers F Rolink A. The status of Ig loci rearrangements in single cells from different stages of B cell development. Int Immunol. (1995) 7:1013–9. 10.1093/intimm/7.6.1013 7577795

[2] Ghia P ten Boekel E Sanz E de la Hera A Rolink A Melchers F. Ordering of human bone marrow B lymphocyte precursors by single-cell polymerase chain reaction analyses of the rearrangement status of the immunoglobulin H and L chain gene loci. J Exp Med. (1996) 184:2217–29. 10.1084/jem.184.6.2217 8976177 PMC2196361

[3] Ghia P Melchers F Rolink AG. Age-dependent changes in B lymphocyte development in man and mouse. Exp Gerontol. (2000) 35:159–65. 10.1016/S0531-5565(99)00095-9 10767576

[4] LeMaoult J Manavalan JS Dyall R Szabo P Nikolic-Zugic J Weksler ME. Cellular basis of B cell clonal populations in old mice. J Immunol. (1999) 162:6384–91. 10352251

[5] Kumar SK Rajkumar V Kyle RA van Duin M Sonneveld P Mateos MV Multiple myeloma. Nat Rev Dis Primers 3:17046. 10.1038/nrdp.2017.46 28726797

[6] Scarfo L Ferreri AJ Ghia P. Chronic lymphocytic leukaemia. Crit Rev Oncol Hematol. (2016) 104:169–82. 10.1016/j.critrevonc.2016.06.003 27370174

[7] Ghia P Prato G Scielzo C Stella S Geuna M Guida G. Monoclonal CD5+ and CD5- B-lymphocyte expansions are frequent in the peripheral blood of the elderly. Blood (2004) 103:2337–42. 10.1182/blood-2003-09-3277 14630808

[8] Scarfo L Dagklis A Scielzo C Fazi C Ghia P. CLL-like monoclonal B-cell lymphocytosis: are we all bound to have it? Semin Cancer Biol. (2010) 20:384–90. 10.1016/j.semcancer.2010.08.005 20816789

[9] Rawstron AC Bennett FL O'Connor SJ Kwok M Fenton JA Plummer M. Monoclonal B-cell lymphocytosis and chronic lymphocytic leukemia. N Engl J Med. (2008) 359:575–83. 10.1056/NEJMoa075290 18687638

[10] Nieto WG Almeida J Romero A Teodosio C Lopez A Henriques AF. Increased frequency (12%) of circulating chronic lymphocytic leukemia-like B-cell clones in healthy subjects using a highly sensitive multicolor flow cytometry approach. Blood (2009) 114:33–7. 10.1182/blood-2009-01-197368 19420353

[11] Shanafelt TD Kay NE Call TG Zent CS Jelinek DF LaPlant B. MBL or CLL: which classification best categorizes the clinical course of patients with an absolute lymphocyte count >or = 5 x 10(9) L(-1) but a B-cell lymphocyte count <5 x 10(9) L(-1)? Leuk Res. (2008) 32:1458–61. 10.1016/j.leukres.2007.11.030 18179821 PMC2587320

[12] Landgren O Albitar M Ma W Abbasi F Hayes RB Ghia P. B-cell clones as early markers for chronic lymphocytic leukemia. N Engl J Med. (2009) 360:659–67. 10.1056/NEJMoa0806122 19213679 PMC7015348

[13] Marti GE Rawstron AC Ghia P Hillmen P Houlston RS Kay N. Diagnostic criteria for monoclonal B-cell lymphocytosis. Br J Haematol. (2005) 130:325–32. 10.1111/j.1365-2141.2005.05550.x 16042682

[14] Rawstron AC Shanafelt T Lanasa MC Landgren O Hanson C Orfao A. Different biology and clinical outcome according to the absolute numbers of clonal B-cells in monoclonal B-cell lymphocytosis (MBL). Cytometry B Clin Cytom. (2010) 78(Suppl. 1):S19–23. 10.1002/cyto.b.20533 20839333 PMC6854899

[15] Fazi C Scarfo L Pecciarini L Cottini F Dagklis A Janus A. General population low-count CLL-like MBL persists over time without clinical progression, although carrying the same cytogenetic abnormalities of CLL. Blood (2011) 118:6618–25. 10.1182/blood-2011-05-357251 21876118

[16] Shanafelt TD Kay NE Jenkins G Call TG Zent CS Jelinek DF. B-cell count and survival: differentiating chronic lymphocytic leukemia from monoclonal B-cell lymphocytosis based on clinical outcome. Blood (2009) 113:4188–96. 10.1182/blood-2008-09-176149 19015397 PMC2676080

[17] Swerdlow SH Campo E Pileri SA Harris NL Stein H Siebert R. The 2016 revision of the World Health Organization classification of lymphoid neoplasms. Blood (2016) 127:2375–90. 10.1182/blood-2016-01-643569 26980727 PMC4874220

[18] Damle RN Wasil T Fais F Ghiotto F Valetto A Allen SL. Ig V gene mutation status and CD38 expression as novel prognostic indicators in chronic lymphocytic leukemia. Blood (1999) 94:1840–7. 10477712

[19] Hamblin TJ Davis Z Gardiner A Oscier DG Stevenson FK. Unmutated Ig V(H) genes are associated with a more aggressive form of chronic lymphocytic leukemia. Blood (1999) 94:1848–54. 10477713

[20] Schroeder HW Jr. Dighiero G. The pathogenesis of chronic lymphocytic leukemia: analysis of the antibody repertoire. Immunol Today (1994) 15:288–94. 10.1016/0167-5699(94)90009-4 7520700

[21] Chiorazzi N Rai KR Ferrarini M. Chronic lymphocytic leukemia. N Engl J Med. (2005) 352:804–15. 10.1056/NEJMra041720 15728813

[22] Rossi D Terzi-di-Bergamo L De Paoli L Cerri M Ghilardi G Chiarenza A. Molecular prediction of durable remission after first-line fludarabine-cyclophosphamide-rituximab in chronic lymphocytic leukemia. Blood (2015) 126:1921–4. 10.1182/blood-2015-05-647925 26276669 PMC4743433

[23] Fischer K Bahlo J Fink AM Goede V Herling CD Cramer P. Long-term remissions after FCR chemoimmunotherapy in previously untreated patients with CLL: updated results of the CLL8 trial. Blood (2016) 127:208–15. 10.1182/blood-2015-06-651125 26486789

[24] Thompson PA Tam CS O'Brien SM Wierda WG Stingo F Keating MJ. Fludarabine, cyclophosphamide, and rituximab treatment achieves long-term disease-free survival in IGHV-mutated chronic lymphocytic leukemia. Blood (2016) 127:303–9. 10.1182/blood-2015-09-667675 26492934 PMC4760129

[25] Hallek M Cheson BD Catovsky D Caligaris-Cappio F Dighiero G Dohner H. Guidelines for diagnosis, indications for treatment, response assessment and supportive management of chronic lymphocytic leukemia. Blood (2018) 131:2745–60. 10.1182/blood-2017-09-806398 29540348

[26] Rosenquist R Ghia P Hadzidimitriou A Sutton LA Agathangelidis A Baliakas P. Immunoglobulin gene sequence analysis in chronic lymphocytic leukemia: updated ERIC recommendations. Leukemia (2017) 31:1477–81. 10.1038/leu.2017.125 28439111 PMC5508071

[27] Stamatopoulos K Agathangelidis A Rosenquist R Ghia P. Antigen receptor stereotypy in chronic lymphocytic leukemia. Leukemia (2017) 31:282–91. 10.1038/leu.2016.322 27811850

[28] Agathangelidis A Darzentas N Hadzidimitriou A Brochet X Murray F Yan XJ. Stereotyped B-cell receptors in one-third of chronic lymphocytic leukemia: a molecular classification with implications for targeted therapies. Blood (2012) 119:4467–75. 10.1182/blood-2011-11-393694 22415752 PMC3392073

[29] Dohner H Stilgenbauer S Benner A Leupolt E Krober A Bullinger L. Genomic aberrations and survival in chronic lymphocytic leukemia. N Engl J Med. (2000) 343:1910–6. 10.1056/NEJM200012283432602 11136261

[30] Puente XS Pinyol M Quesada V Conde L Ordonez GR Villamor N. Whole-genome sequencing identifies recurrent mutations in chronic lymphocytic leukaemia. Nature (2011) 475:101–5. 10.1038/nature10113 21642962 PMC3322590

[31] Wang L Lawrence MS Wan Y Stojanov P Sougnez C Stevenson K. SF3B1 and other novel cancer genes in chronic lymphocytic leukemia. N Engl J Med. (2011) 365:2497–506. 10.1056/NEJMoa1109016 22150006 PMC3685413

[32] Rossi D Fangazio M Rasi S Vaisitti T Monti S Cresta S. Disruption of BIRC3 associates with fludarabine chemorefractoriness in TP53 wild-type chronic lymphocytic leukemia. Blood (2012) 119:2854–62. 10.1182/blood-2011-12-395673 22308293

[33] Rosenquist R Rosenwald A Du MQ Gaidano G Groenen P Wotherspoon A. Clinical impact of recurrently mutated genes on lymphoma diagnostics: state-of-the-art and beyond. Haematologica (2016) 101:1002–9. 10.3324/haematol.2015.134510 27582569 PMC5060016

[34] Herishanu Y Perez-Galan P Liu D Biancotto A Pittaluga S Vire B. The lymph node microenvironment promotes B-cell receptor signaling, NF-kappaB activation, and tumor proliferation in chronic lymphocytic leukemia. Blood (2011) 117:563–74. 10.1182/blood-2010-05-284984 20940416 PMC3031480

[35] Herve M Xu K Ng YS Wardemann H Albesiano E Messmer BT. Unmutated and mutated chronic lymphocytic leukemias derive from self-reactive B cell precursors despite expressing different antibody reactivity. J Clin Invest. (2005) 115:1636–43. 10.1172/JCI24387 15902303 PMC1088018

[36] Lanemo Myhrinder A Hellqvist E Sidorova E Soderberg A Baxendale H Dahle C. A new perspective: molecular motifs on oxidized LDL, apoptotic cells, and bacteria are targets for chronic lymphocytic leukemia antibodies. Blood (2008) 111:3838–48. 10.1182/blood-2007-11-125450 18223168

[37] Duhren-von Minden M Ubelhart R Schneider D Wossning T Bach MP Buchner M. Chronic lymphocytic leukaemia is driven by antigen-independent cell-autonomous signalling. Nature (2012) 489:309–12. 10.1038/nature11309 22885698

[38] Kohler F Hug E Eschbach C Meixlsperger S Hobeika E Kofer J. Autoreactive B cell receptors mimic autonomous pre-B cell receptor signaling and induce proliferation of early B cells. Immunity (2008) 29:912–21. 10.1016/j.immuni.2008.10.013 19084434

[39] Minici C Gounari M Ubelhart R Scarfo L Duhren-von Minden M Schneider D. Distinct homotypic B-cell receptor interactions shape the outcome of chronic lymphocytic leukaemia. Nat Commun. (2017) 8:15746. 10.1038/ncomms15746 28598442 PMC5472768

[40] Muzio M Apollonio B Scielzo C Frenquelli M Vandoni I Boussiotis V. Constitutive activation of distinct BCR-signaling pathways in a subset of CLL patients: a molecular signature of anergy. Blood (2008) 112:188–95. 10.1182/blood-2007-09-111344 18292287

[41] Byrd JC Brown JR O'Brien S Barrientos JC Kay NE Reddy NM. Ibrutinib versus ofatumumab in previously treated chronic lymphoid leukemia. N Engl J Med. (2014) 371:213–23. 10.1056/NEJMoa1400376 24881631 PMC4134521

[42] Furman RR Sharman JP Coutre SE Cheson BD Pagel JM Hillmen P. Idelalisib and rituximab in relapsed chronic lymphocytic leukemia. N Engl J Med. (2014) 370:997–1007. 10.1056/NEJMoa1315226 24450857 PMC4161365

